# ^18^F-FLT-PET/CT adds value to ^18^F-FDG-PET/CT for diagnosing relapse after definitive radiotherapy in patients with lung cancer. Results of a prospective clinical trial

**DOI:** 10.2967/jnumed.120.247742

**Published:** 2020-10-09

**Authors:** Tine Nøhr Christensen, Seppo W Langer, Gitte Persson, Klaus Richter Larsen, Annika Loft, Annemarie Gjelstrup Amtoft, Anne Kiil Berthelsen, Helle Hjorth Johannesen, Sune Høgild Keller, Andreas Kjaer, Barbara Malene Fischer

**Affiliations:** 1 Department of Clinical Physiology, Nuclear Medicine & PET, Copenhagen University Hospital, Rigshospitalet, Denmark; 2 Cluster for Molecular Imaging, University of Copenhagen, Denmark; 3 Department of Oncology, Copenhagen University Hospital, Rigshospitalet, Denmark; 4 Department of Oncology, Herlev-Gentofte Hospital, University of Copenhagen, Denmark; 5 Department of Clinical Medicine, Faculty of Health, University of Copenhagen, Denmark; 6 Department of Pulmonary Medicine, Bispebjerg University Hospital, Denmark; 7 The PET Centre, School of Biomedical Engineering and Imaging Science, King’s College London, UK

**Keywords:** Lung cancer, Radiotherapy, Relapse, FDG-PET/CT, FLT-PET/CT

## Abstract

Diagnosing relapse after radiotherapy for lung cancer is challenging. The specificity of both CT and 2-deoxy-2-[^18^F]fluoro-D-glucose (FDG)-PET/CT is low due to radiation-induced changes. 3’-deoxy-3’-[^18^F]fluorothymidine (FLT)-PET has previously demonstrated higher specificity for malignancy than FDG-PET. We investigated the value of FLT-PET/CT for diagnosing relapse in irradiated lung cancer.

**Methods:**

Patients suspected for relapse of lung cancer after definitive radiotherapy (conventional fractionated radiotherapy (cRT) or stereotactic radiotherapy (SBRT)) were included.

Sensitivity and specificity were analysed within the irradiated high-dose volume (HDV) and patient-based.

Marginal differences and inter-observer agreement were assessed.

**Results:**

Sixty-three patients who had received radiotherapy in 70 HDVs (34 cRT; 36 SBRT) were included.

The specificity of FLT-PET/CT was higher than FDG-PET/CT (HDV: 96% [87-100] vs. 71% [57-83]; p=0.0039; patient-based (90 % [73-98] vs. 55% [36-74]; p=0.0020)). The difference between specificity of FLT-PET/CT and FDG-PET/CT was higher after cRT compared with SBRT. Sensitivity of FLT-PET/CT was lower than FDG-PET/CT (HDV: 69% [41-89] vs. 94% [70-100]; p=0.1250; patient-based: 70% [51-84] vs. 94% [80-99]; p=0.0078).

Adding FLT-PET/CT when FDG-PET/CT was positive or inconclusive improved diagnostic value compared with FDG-PET/CT only. In cRT-HDVs, the probability of malignancy increased from 67% for FDG-PET/CT alone to 100% when both PETs were positive.

**Conclusion:**

FLT-PET/CT adds diagnostic value to FDG-PET/CT in patients with suspected relapse. The diagnostic impact of FLT-PET/CT was highest after cRT. We suggest adding FLT-PET/CT when FDG-PET/CT is inconclusive or positive within the previously irradiated volume to improve diagnostic value in patients where histological confirmation is not easily obtained.

## Introduction

Disease control after definitive radiotherapy is initially high, but 15-40% of the patients will eventually experience loco-regional failure (1–5). Many patients experience radiation-induced pneumonitis (3-35%) and/or fibrosis (30-50%) after radiotherapy (3,6) and distinguishing local recurrence from radiation-induced lung injuries is challenging. Active surveillance with CT is recommended (7), but changes on CT after radiotherapy may mimic recurrence (8). 2-deoxy-2-[^18^F]fluoro-D-glucose (FDG-)PET/CT is recommended if relapse is suspected (9). Post-treatment inflammation may however cause high FDG-uptake, thus reducing the specificity of FDG-PET (10).

3’-deoxy-3’-[^18^F]fluorothymidine (FLT) is a marker of proliferation (11). FLT-PET has a higher specificity than FDG-PET and performed better in differential diagnosis of inflammatory lesions in lung lesions (12). The potential of FLT-PET to differentiate malignancy from radiation-induced changes is less well described (13–15).

One small study showed correct diagnosis of disease progression with FLT-PET/CT in seven of eight patients after stereotactic body irradiation (SBRT) for lung cancer (14). To our knowledge, no publications have addressed the diagnostic value of FLT-PET after conventional fractionated radiotherapy (cRT) in patients with lung cancer.

In the current study, we hypothesized that FLT-PET/CT could improve relapse diagnosis after radiotherapy for lung cancer.

## Materials and Methods

### Patients

Patients were prospectively included if meeting the following inclusion criteria: Histologically confirmed non-small cell lung cancer (NSCLC) or small cell lung cancer (SCLC), treatment with definitive radiotherapy within the last 24 months and current suspicion of relapse warranting an FDG-PET/CT. The cause of relapse suspicion is specified in Table 1. Patients were analyzed according to treatment regime: 1) cRT i.e. normo- and hyperfractionated radiotherapy, and 2) SBRT.

Patients were recruited from Copenhagen University Hospital, Rigshospitalet, Bispebjerg University Hospital and Herlev University Hospital in Denmark from January 2015 to January 2019. The study protocol was approved by the local Ethics Committee (approval number H-4-2014-060) and by institutional review boards. All patients signed a written informed consent. The study was registered at clinicaltrials.gov (ID: NCT029995889).

### Imaging

FDG-PET/CT was conducted as a routine clinical investigation at the referring hospital according to local procedures. Details are available in Supplemental Table 1. Patients fasted at least 4 hours before injection of FDG (200 Mbq or 4 Mbq/kg according to institutional protocol) and rested 60 minutes between injection and scan. Images were reconstructed following vendor recommendations and/or international clinical guidelines for FDG-PET imaging.

FLT-PET/(low-dose-)CT was performed at Rigshospitalet on a Siemens Biograph TruePoint TrueV 40 or 64 PET/CT-scanner. FLT (5 MBq/kg, max 350 MBq) was injected 60±10 minutes prior to PET/CT without restrictions regarding fasting or resting. Static regional imaging was obtained from the skull base to the iliac bone. FLT-PET images were reconstructed using ordered subset expectation maximization (OSEM) with point spread function modelling (PSF), 3 iterations and 21 subsets with a 2 mm full width half maximum (FWHM) Gaussian post-reconstruction filter.

### Image Analysis

All images were analyzed on a Mirada Medical Ltd. XD 3.6 workstation.

PET/CT were interpreted retrospectively as project readings, independently of subsequent management of the patients, blinded for clinical data and previous PET-scans, but not previous CTs. Project readings were performed qualitatively and jointly by an experienced nuclear medicine physician and radiologist. FLT-PET/CTs were double read by two observer-teams. Interpretation of FDG-PET/CT and FLT-PET/CT from the same patient by the same observer-team was restricted to a time lapse of minimum three months.

Up to three lesions in each PET/CT were evaluated for malignancy using a 5-point scale: definitely benign, probably benign, inconclusive, probably malignant, and definitely malignant (patient-based analysis). From the previous radiotherapy-plan, the high-dose volume (HDV) was defined within the 50% isodose curve, and PET-evaluated lesions within HDV were identified (HDV-based analysis). If an HDV-lesion was not matched with a PET-evaluated lesion, the HDV-lesion was given the evaluation “definitely benign”.

SUV_max_ from FDG-PET and FLT-PET was measured in the evaluated lesions and in HDV.

### Endpoint And Reference Standard

The endpoint was relapse status (relapse/no relapse) within six months after FLT-PET/CT. Confirmation by histology was encouraged in the protocol. However, if not clinically feasible a compound reference standard for was applied. This was assigned by an experienced clinical oncologist based on review of patient records including histology, imaging, invasive procedures, and conference decisions. The clinical oncologist was blinded for name and age of the patient, dates, and name of involved physicians.

### Statistics

Study size was determined from a power calculation based on previous studies suggesting different results from FDG-PET and FLT-PET in at least 20% of lung cancer patients (16–18). With a power of 80%, and a two-sided alfa-level of significance of 0.05, the study population should be at least 29 patients in each group. Taking the possibility of drop-outs into account, each group was appointed up to 35 patients.

The diagnostic values of FDG-PET/CT and FLT-PET/CT were analyzed within HDV and patient-based as a whole-body analysis. For HDV-based analysis, all HDVs from each patient were included. For the patient-based analysis, the worst grading on the 5-point scale in each patient were selected. Sensitivity, specificity, negative predictive value (NPV), positive predictive value (PPV), and accuracy were calculated. Inconclusive PET-results were included in the analysis one time as positive result and one time as a negative result, and the two scenarios were analyzed separately. Patients/HDVs with inconclusive reference standard were excluded from the diagnostic analysis. Marginal differences of sensitivity and specificity of FDG-PET/CT vs. FLT-PET/CT were calculated by McNemar tests for all four combinations of handling inconclusive FDG-PET- and FDG-PET-results. Inter-observer agreement was calculated with kappa statistics for positive vs. negative and inconclusive FLT-PET/CT.

A model combining FDG-PET/CT and FLT-PET/CT was suggested, and the diagnostic value of combined FDG-PET/CT and FLT-PET/CT was calculated.

Statistical analyses were performed in SPSS, version 25. 95% confidence intervals (95% CI) for diagnostic value and marginal differences were performed using MedCalc, version 19.2 (MedCalc Software Ltd, Ostend, Belgium).

## Results

### Patients

**Figure 1:**
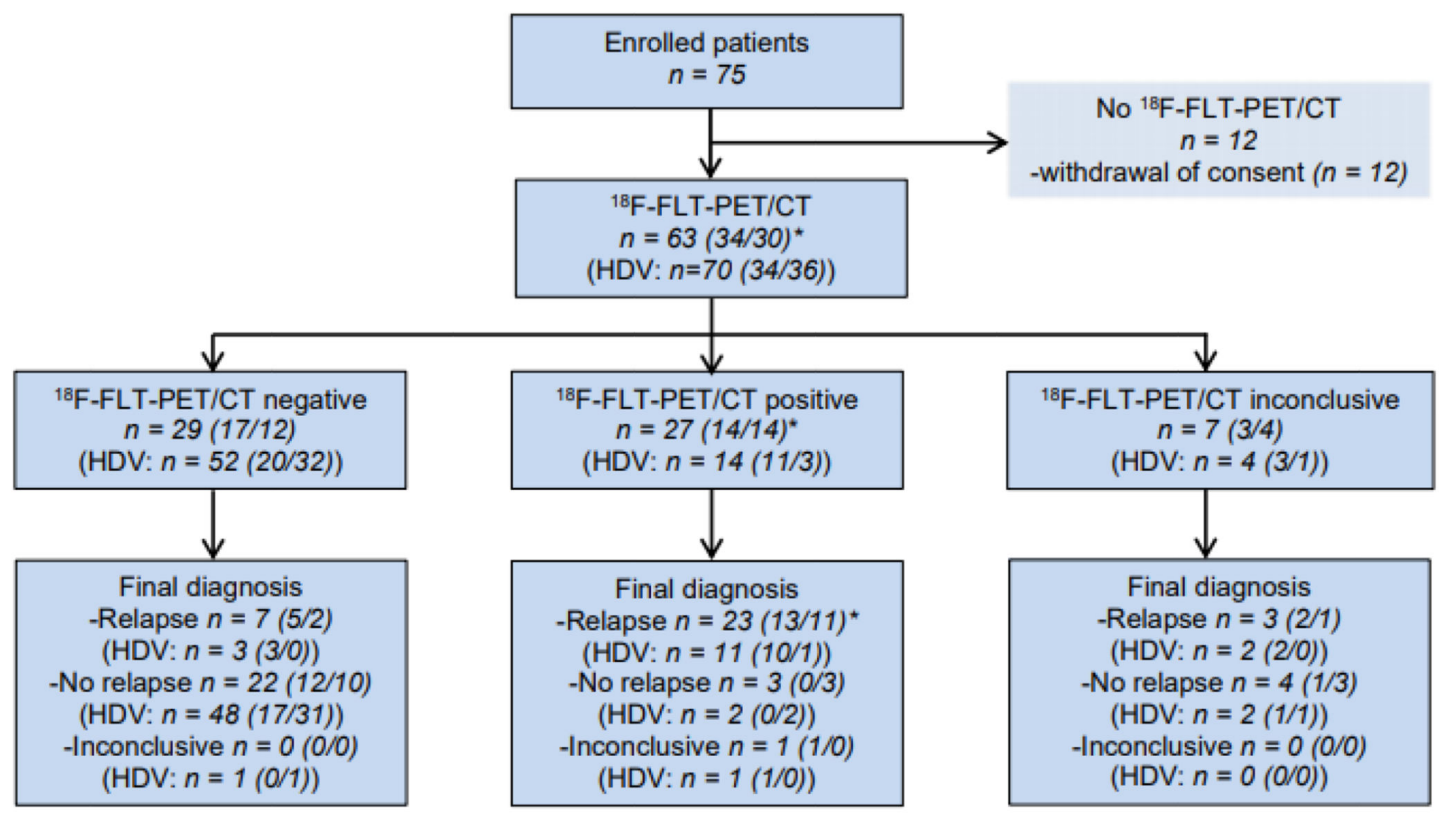
Patient flow in the study. Numbers in brackets refer to the subgroup (conventional fractionated radiotherapy/stereotactic radiotherapy). *One patient was included in both subgroups. HDV: irradiated high-dose volume.

A total of 75 patients were enrolled; however, 12 patients withdraw consent, thus 63 patients were evaluable, Figure 1. Two patients participated twice in the study; second time due to a new suspicion of relapse. One patient received cRT *and* SBRT and was included in both subgroups. This patient was initially treated with cRT, and later with SBRT due to a new malignant lesion. Accordingly, 34 patients had been treated with cRT and 30 patients with SBRT. In accordance with the indications for the radiotherapy-regimes, patients in the cRT-group had a higher stage at time of diagnosis than the SBRT-patients. Patient characteristics are presented in Table 1.

A total of 70 HDVs (34 cRT-HDVs; 36 SBRT-HDVs) from the 63 patients were included in the analysis: Two patients were treated with radiotherapy twice; the second time due to local relapse. Four patients received SBRT in two (n=3) or three SBRT-HDVs (n=1) at initial diagnosis due to several lung lesion. In each patient, cRT-HDV was coherent, thus one cRT-HDV was included per cRT-patient.

### Diagnostic Value of FDG-PET/CT And FLT-PET/CT in Irradiated High-Dose Volume

During the six months’ follow-up, relapse was diagnosed in 16 HDVs. Relapse in HDV was confirmed by biopsy or subsequent progression in 10/16 HDVs. Non-relapse was confirmed by 6 months follow-up without progression or by negative biopsy in 45/52 HDVs. In the remaining HDVs confirmation level was low, Table 2.

FLT-PET/CT and FDG-PET/CT was positive in 14 respectively 29 HDVs. Sensitivity and NPV were lower for FLT-PET/CT than for FDG-PET/CT, and the specificity and PPV were higher for FLT-PET/CT than for FDG-PET/CT both when considering inconclusive PET-results positive as well as when considering inconclusive PET-results negative. Results from all diagnostic analysis are presented in Table 3. Cross-tabulation of PET-results relative to clinical outcome are available in Supplemental Table 2.

For simplification this and the following subsection describe results from analyses considering inconclusive FDG-PET/CT-results as positive and inconclusive FLT-PET/CT-results as negative.

The specificity of FLT-PET/CT within HDV was 25 percentage points [95% CI: 13-37] higher than the specificity of FDG-PET/CT (p=0.0039), i.e. FDG-PET/CT was false positive in 25% more cases than FLT/PET/CT. The difference of the specificity was largest in the cRT-HDVs (cRT-HDV: 39 percentage points [95% CI: 16-61]; p=0.0156; SBRT-HDV: 18 percentage points [95% CI: 5-30]; p=0.0313).

Though the sensitivity of FDG-PET/CT was higher than the sensitivity of FLT-PET/CT, the difference was not significant (all: 25 percentage points [95% CI: 4-46]; p=0.1250; cRT-HDV: 27 percentage points [95% CI: 4-49]; p=0.1250; SBRT-HDV: inconclusive due to only one relapse). Cross-tabulations of FLT-PET-results vs. FDG-PET-results and results from all McNemar analysis with variant handlings of inconclusive results are available in Supplemental Table 3 and 4.

FLT-SUV_max_ in relapsed HDVs was 1.8-9.7 (median: 2.4) compared with 0.4-4.5 (median: 2.2) in benign HDVs. FDG-SUV_max_ in relapsed HDVs was 4.0-20.5 (median: 12.8) compared with 0.7-17.5 (median: 4.1) in benign HDVs.

PET-images illustrating the diagnostic strengths and weaknesses are shown in Figure 3.

### Patient-Based Diagnostic Value of FDG-PET/CT And FLT-PET/CT

**Figure 2:**
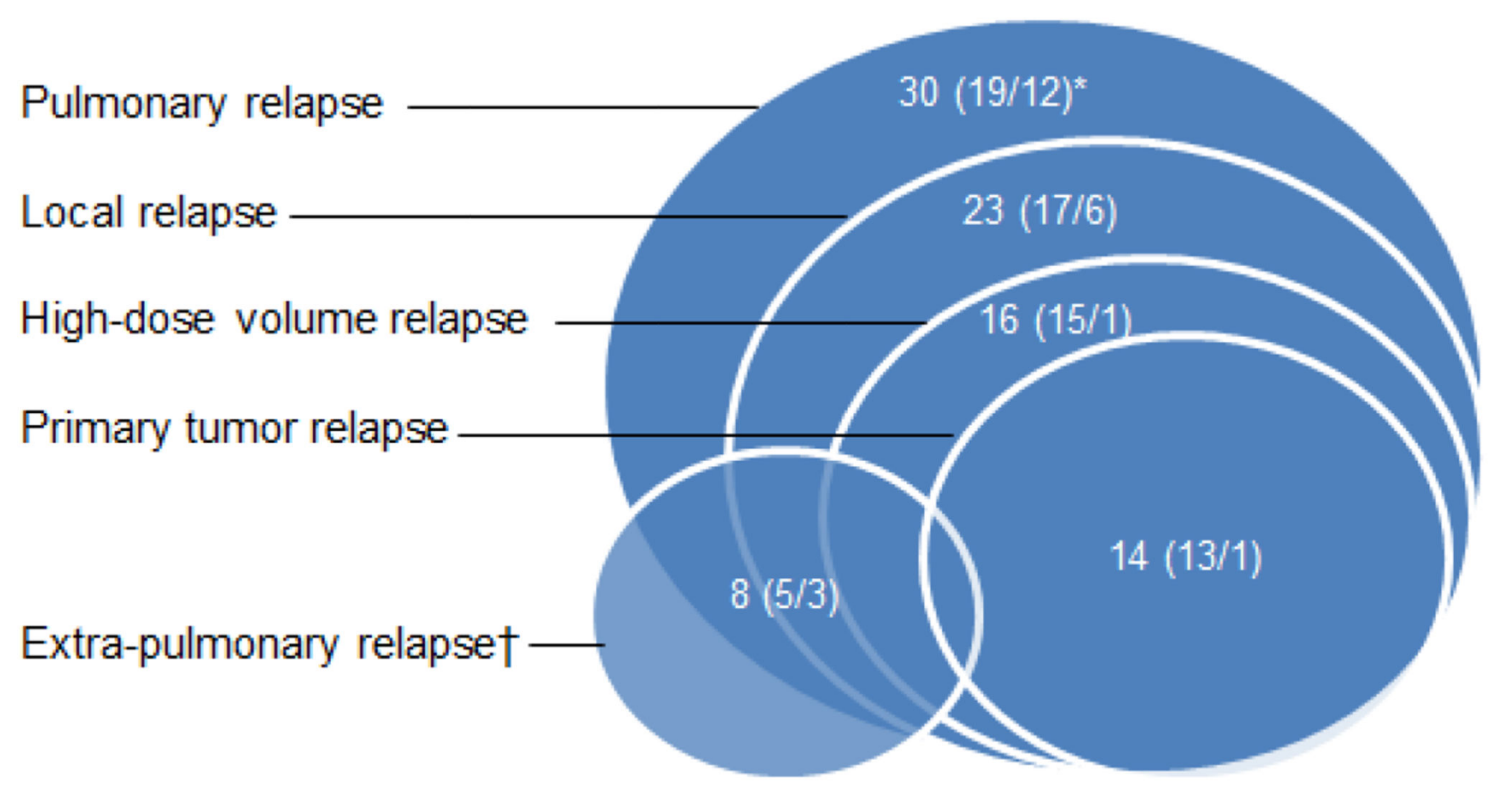
Location of relapse. A total of 33 patients had relapse; of these 30 patients had pulmonary relapse. Numbers in brackets refer to the subgroups (conventional fractionated radiotherapy/stereotactic radiotherapy). *One patient was included in both subgroups; †3 patients (1/2) had only extra-pulmonary relapse

**Figure 3:**
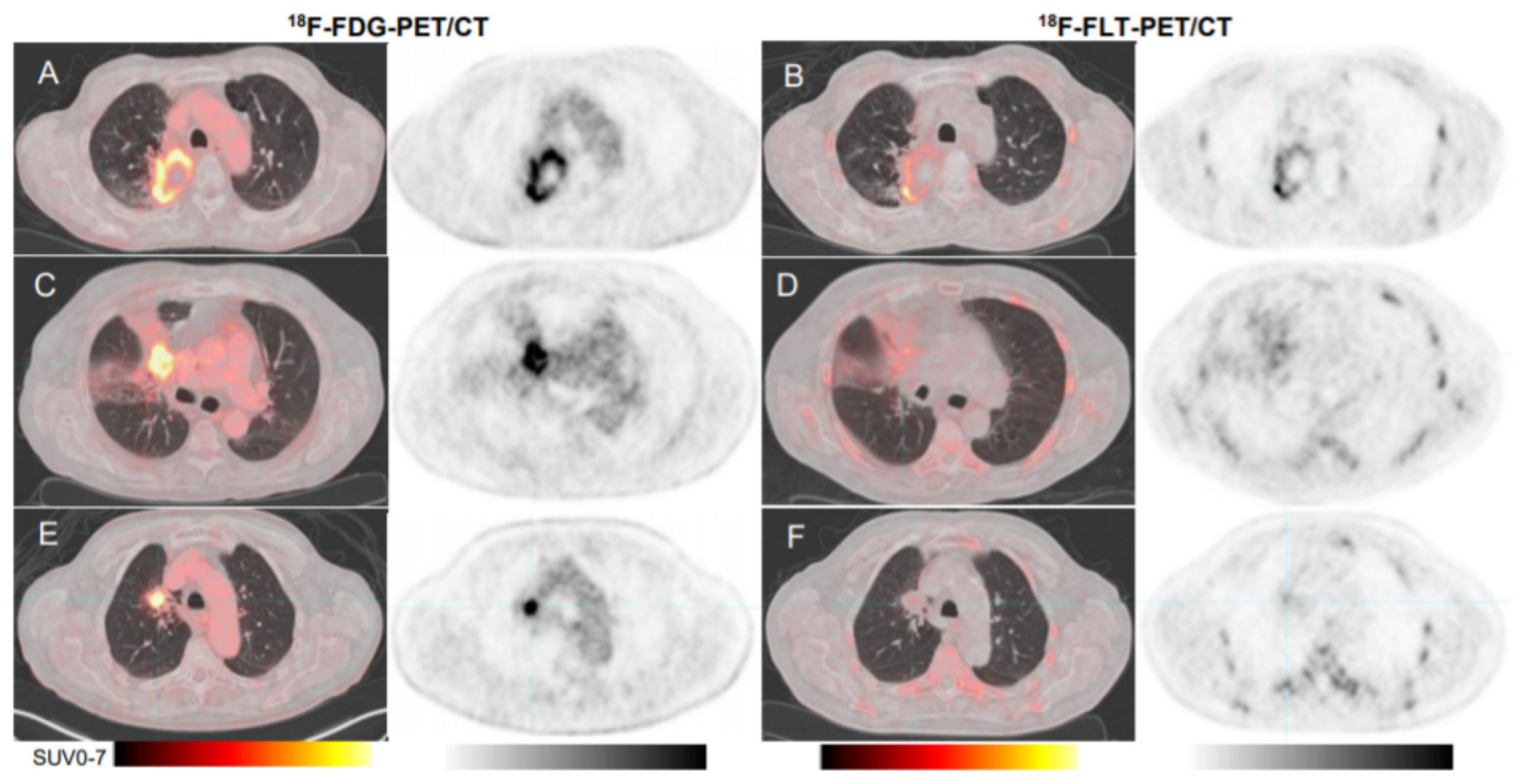
FDG-PET/CT and FLT-PET/CT in three representative patients with suspected relapse after conventional fractionated radiotherapy (cRT) of a lung cancer. A-B: Relapse 19 months after end of cRT detected by FDG-PET/CT (A) and FLT-PET/CT (B). C-D: No relapse 4 months after end of cRT. FDG-PET/CT (C) was false positive. FLT-PET/CT (D) was true negative. E-F: Relapse 15 months after end of cRT. FDG-PET/CT (E) was true positive. FLT-PET/CT (F) was false negative. The relapse was located in lung tissue as confirmed by biopsy, not in lymph node as it may appear on these examinations.

During follow-up, 33 patients (52%) were diagnosed with relapse. Figure 2 illustrates the location of the relapse. In 19/33 patients, relapse was confirmed by biopsy or subsequent progression according to RECIST 1.1. Non-relapse was confirmed by 6 months follow-up without progression according to RECIST 1.1 in all patients, Table 2.

FLT-PET/CT and FDG-PET/CT was positive in 27 respectively 43 patients. Cross-tabulations and diagnostic value are available in Supplemental Table 2 and 5.

The specificity of FLT-PET/CT was 34 percentage points [95% CI: 17-52] higher than the specificity of FDG-PET/CT in all patients (90% [95% CI: 73-98] vs. 55% [95% CI: 36-74]; p=0.0020). In cRT-patients, FLT-PET/CT outperformed FDG-PET/CT with a 54 percentage points [95% CI: 27-81] higher specificity (100% [95% CI: 75-100] vs. 46% [95% CI: 19-75]; p=0.0156). The specificity was not significantly different in SBRT-patients (19 percentage points [95% CI: -0.4-38]; p=0.2500).

The sensitivity of FDG-PET/CT was 24 percentage points [95% CI: 10-39] higher than the sensitivity of FLT-PET/CT in all patients (94% [95% CI: 80-99] vs. 70% [95% CI: 51-84], p=0.0078). In the subgroups, the difference of the sensitivity did not reach statistical significance (cRT-patients: 25 percentage points [95% CI: -6-44]; p=0.0625; SBRT-patients: 21 percentage points [95% CI: -0.1-43]; p=0.2500). Cross-tabulations and McNemar analysis with variant handlings of inconclusive PET-results are available in Supplemental Table 3 and 6.

FLT-SUV_max_ in patients with pulmonary relapse was 0.9-9.7 (median 3.7) compared with 0.8-4.5 (median 2.5) in patients without pulmonary relapse, measured in the worst graded lesions from the 5-point grading scale. FDG-SUV_max_ in patients with pulmonary relapse was 1.2-20.5 (median 8.6) compared with 1.9-17.5 (median: 4.6) in patients without pulmonary relapse.

### Inter-Observer Agreement of FLT-PET/CT

Patient-based and HDV-based inter-observer agreement was moderate (0.47 and 0.57). Inter-observer agreement was highest in cRT-patients (0.68) and cRT-HDVs (0.70) and only moderate or poor in SBRT-patients (0.45) and SBRT-HDVs (-0.04).

### Combined Diagnostic Value of FDG-PET/CT And FLT-PET/CT

**Figure 4:**
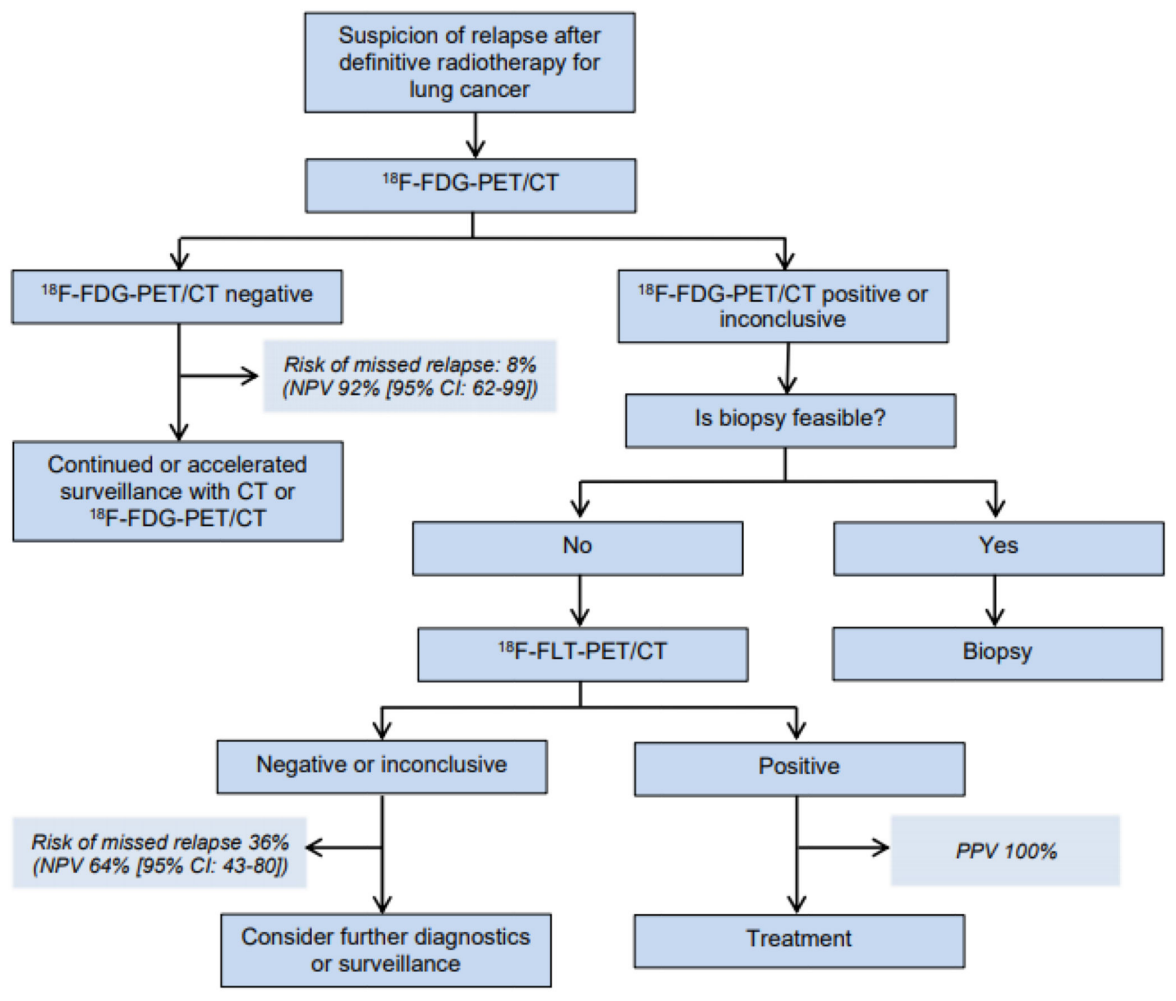
Suggested diagnostic flow for patients suspected for having a relapse within the irradiated high-dose volume. Positive predictive value (PPV) and negative predictive value (NPV) are given for high-dose volumes treated with conventional fractionated radiotherapy.

To exploit the high NPV of FDG-PET/CT and the high PPV of FLT-PET/CT, we suggest adding FLT-PET/CT when FDG-PET/CT is positive or inconclusive. When FDG-PET/CT was negative, FLT-PET/CT provided no additional value, as all negative FDG-PET/CT-results were accompanied by negative FLT-PET/CT-results. The suggested diagnostic flow is illustrated in Figure 4.

Diagnostic accuracy was improved in the combined model compared with a single positive/inconclusive FDG-PET/CT, Table 4. The impact of adding FLT-PET/CT to positive/inconclusive FDG-PET/CT was highest in cRT-patients, raising the probability of malignancy from 72% after positive/inconclusive FDG-PET/CT, to 100% when FLT-PET/CT was positive.

## Discussion

The main finding of this study is that FLT-PET/CT with a high specificity and PPV adds value to FDG-PET/CT for the detection of relapse of lung cancer after radiotherapy. The sensitivity of FLT-PET/CT was in most settings not significantly different from that of FDG-PET/CT.

The superior specificity of FLT-PET/CT is consistent with results from pretreatment studies (12) and results after SBRT (14). In the small study of Hiniker et al. sensitivity (80% (4/5)) and specificity (100% (3/3)) were high with only one false negative FLT-PET/CT after SBRT for lung cancer (14). Hiniker et al. included patients with suspicious FDG-PET/CTs, and the rate of local relapse was higher than in our study (5/8 vs. 1/35). With only one SBRT-HDV relapse, our study did not have statistical power to conclude on the sensitivity in this group. Similar results have also been demonstrated after concomitant chemo-radiotherapy for esophageal cancer; FLT-PET/CT was superior to FDG-PET/CT at distinguishing malignant tissue from esophagitis (13).

We demonstrated a higher difference of the specificity of FLT-PET/CT vs. FDG-PET/CT after cRT compared with SBRT caused by a combination of lower specificity for FDG-PET/CT and higher specificity of FLT-PET/CT after cRT compared with SBRT. Different patterns of injuries in the surrounding lung tissue from different radiotherapy-regimes (3,6) may explain this difference. Toxicity is related to dose deposited in surrounding lung tissues; larger HDV in cRT-regimes cause larger volumes of lung tissue to be exposed. Smaller HDV from SBRT-regimes spares the surrounding lung tissue in higher extend. A higher prevalence of radiation-induced changes may explain the lower specificity of FDG-PET/CT after cRT. The lower specificity and PPV of FLT-PET/CT in the SBRT-HDVs compared with the cRT-HDVs might be caused by the very low prevalence of relapse in SBRT-HDVs.

The difference of the specificity of FDG-PET/CT and FLT/CT was higher on patient-basis than within HDV, as a result of the low specificity of FDG-PET/CT on a patient-basis. FDG-PET and FLT-PET were evaluated blinded to make comparable and unbiased evaluations. However, in the clinical setting knowledge of previous treatment and possible inflammatory sites is essential for evaluation of FDG-PET/CT (10), and blinded reading might have higher impact on FDG-PET/CT than FLT-PET/CT. With several lesions evaluated in each patient on the patients-based analysis, the consequence of the blinding was more pronounced on patient-basis than in the HDV-based analysis. To quantify the consequence of the blinding, we compared the blinded FDG-PET/CT-results with results from the clinical FDG-PET/CT-report. The specificity of FDG-PET/CT was 10 percentage points [95% CI: - 12-32] higher in the clinical report compared with the blinded results, but the difference was not significant (p=0.549). Blinding did not affect the sensitivity (94%; p=1). Using FLT-PET in a patient-based analysis is controversial, as FLT-PET has limited use for diagnosing distant metastases due to high background-uptake in the liver and bone (19), and false positive results in lymph nodes may be caused by proliferative B-lymphocytes (20). Our project was not designed to investigate the diagnostic value of FLT-PET/CT on metastases; however, eight patients were diagnosed with metastases in bones or liver. In five patients, FLT-PET/CT missed bone or liver metastases, but due to other malignant lesions detected by FLT-PET/CT, only three patients had false negative FLT-PET/CT due to distant metastases. Accordingly, extra-pulmonary metastases had no impact on specificity in this study, but some impact on patient-based sensitivity.

There were some limitations to our study. Patients with a high suspicion of recurrence could be referred directly for biopsy or oncological treatment, and thus not included in this project. Blinded reading of PET-scans is a deviation from clinical guidelines (10), but was applied to make FDG-PET/CT and FLT-PET/CT evaluations comparable. Combining FDG-PET with diagnostic CT and FLT-PET with low-dose CT, potentially gave FDG-PET/CT an advantage over FLT-PET/CT. Project readings were, however, not blinded for CT and therefore previous diagnostic CTs could be accessed. Combining an added FLT-PET with low-dose CT seems sufficient, and reduces excessive ionizing irradiation and cost. Neither FDG- or FLT-PET/CT was done with respiratory gating. This could potentially lead to a mis-registration between PET and CT and a potential underestimation of tracer uptake, especially in small nodules. Relapse status was in most cases confirmed by either histology or follow-up with subsequent progression or non-progression. In some patients, relapse diagnosis was based solely on FDG-PET/CT, as decided by multi-disciplinary conference, due to an obvious outcome of FDG-PET/CT and/or the patient being unfit for invasive procedures. FDG-PET/CT is recommended as second-step test for patients with suspected relapse after radiotherapy (7), and therefore we did not exclude patients without further confirmation than FDG-PET/CT. Thus, in these cases, the test result of FDG-PET/CT and the reference was not independent; potentially overestimating sensitivity and specificity of FDG-PET/CT. When FLT-PET/CT and FDG-PET/CT-results were in agreement, a potential overestimate would concern the absolute values of sensitivity and specificity of FLT-PET/CT and FDG-PET/CT, but not their differences. However, when FLT-PET/CT-results and FDG-PET/CT-results were not in agreement, and the reference was based solely on FDG-PET/CT, FLT-PET/CT-results would always be false. Only in two patients in whom relapse was based solely on the FDG-PET/CT, were FLT-PET/CT-results not in agreement with FDG-PET/CT-results. Despite favoring FDG-PET/CT when further confirmation of relapse status was not obtained, the specificity of FLT-PET/CT was significantly higher than FDG-PET/CT.

Early and precise diagnosis of lung cancer relapse is essential, as surgery or re-irradiation with curative intent might be feasible (1). To improve relapse diagnosing, we suggest adding FLT-PET/CT, when FDG-PET/CT is positive or inconclusive within the HDV. We acknowledge that in many cases renewed biopsy is required due to the possibility of pathological transition that potentially changes the treatment of choice. When biopsy is feasible and favored, FLT-PET/CT does not outperform invasive procedures; however, FLT-PET might have a place for guiding biopsies, but further investigations are needed. In the many patients in whom biopsy is not feasible due to poor lung condition or difficult location, FLT-PET/CT adds valuable diagnostic information.

## Conclusion

FLT-PET/CT has a higher specificity than FDG-PET/CT in patients who have been treated with radiotherapy, both within HDV and on a patient-basis. The diagnostic impact of FLT-PET/CT was highest after cRT.

We suggest adding FLT-PET/CT when FDG-PET/CT is inconclusive or positive within HDV in patients who are unfit for invasive procedures, and when renewed histology is not essential for the further course.

## Supplementary Material

Supplementary Material

## Figures and Tables

**Table 1:** Patient characteristics

		All patients (n=63)	cRT-patients (n=34)*	SBRT-patients (n=30)*
Age at FLT-PET/CT (median [range])		70 [55-86]	68 [58-86]	75 [55-86]
Sex (male/female)		36/27	18/16	19/11
Histology				
Adenocarcinoma		30 (47.6%)	15 (44.1%)	15 (50%)
Squamous cell carcinoma		25 (39.7%)	13 (38.2%)	13 (43.3%)
NSCLC not otherwise specified		4 (6.3%)	2 (5.9%)	2 (6.7%)
SCLC		2 (3.2%)	2 (5.9%)	0
Mixed NSCLC/SCLC		2 (3.2%)	2 (5.9%)	0
Stage at diagnosis				
Ia		13 (20.6%)	0	13 (43.3%)
Ib		6 (9.5%)	1 (2.9%)	5 (16.7%)
IIa		2 (3.2%)	0	2 (6.7%)
IIb		5 (7.9%)	1 (2.9%)	4 (13.3%)
IIIa		15 (23.8%)	14 (41.2%)	2 (6.7%)
IIIb		16 (25.4%)	15 (44.1%)	1 (3.3%)
IV		6 (9.5%)	3 (8.8%)	3 (10.0%)
Radiotherapy				
Normofractionated	60 Gy (24-33F)	30 (47.6%)	31 (91.2%)	
Hyperfractionated	45-60 Gy (30-40F)	3 (4.8%)	3 (8.8%)	
SBRT	50 Gy (5F)	2 (3.2%)		2 (6.7%)
	45-72 Gy (3F)	28 (44.4%)		28 (93.3%)
Chemotherapy		35 (55.6%)	32 (94.1%)	3 (10%)
Cause of relapse suspicion				
Symptoms		1 (1.6%)	1 (2.9%)	0
CT (surveilance)		53 (84.1%)	30 (88.2%)	24 (80%)
CT and symptoms		2 (3.2%)	2 (5.9%)	0
FDG-PET/CT (surveillance)		6 (9.5%)	0	6 (20%)
FDG-PET/CT and symptoms		1 (1.6%)	1 (2.9%)	0
Days between radiotherapy end and FLT-PET/CT (median [range])		237 [34-729]	277 [34-626]	236 [108-729]
Days between FDG- and FLT-PET/CT (median [range])		6 [1-30]	6 [1-22]	6 [1-30]

* One patient was included in both subgroups. cRT: conventional fractionated radiotherapy; SBRT: stereotactic radiotherapy; NSCLC: non-small-cell lung cancer; SCLC: small-cell lung cancer; Gy: gray; F: fractions

**Table 2:** Clinical outcome and basis for confirmation

Outcome	Confirmation basis	all HDVs (n=70)	cRT-HDVs (n=34)	SBRT-HDVs (n=36)
Relapse		16	15	1
	Histology	4	4	0
	Subsequent progression	6	5	1
	FDG-PET/CT only	6	6	0
No relapse		52	18	34
	No subsequent progression	44	15	29
	Neg. biopsy (follow-up not applicable due to systematic treatment)	1	0	1
	FDG-PET/CT only (follow-up not applicable due to systematic treatment)	7	3	4
Inconclusive		2† ‡	1†	1‡
		**All patients (n=63)**	cRT-patients (n=34)*	SBRT-patients (n=30)*
Relapse		33	20	14
	Histology	8	4	4
	Subsequent progression	11	5	7
	Disseminated disease	6	4	2
	FDG-PET/CT only	8	7	1
No relapse		29	13	16
	No subsequent progression	29	13	16
Inconclusive		1†	1†	0

* One patient was included in both subgroups;

† biopsies were performed twice; both were suspicious but not conclusive of malignancy. Two months after end of follow-up, relapse was diagnosed based on metastatic adenocarcinoma cells in exudate from the pericardium;

‡ The clinical PET-report described “progression of radiation-induced changes”, and biopsy was suggested, though not performed. Follow-up was not applicable, as the patient received systemic treatment due to a distant relapse.

cRT: conventional fractionated radiotherapy; SBRT: stereotactic radiotherapy; HDV: high-dose volume

**Table 3:** Diagnostic value of FDG-PET/CT and FLT-PET/CT within the irradiated high-dose volume (HDV). Inconclusive PET-results were handled as positive, respectively, negative. Results from blinded PET-evaluations. 95% confidence interval in square brackets.

	PET	Handling of inconclusive PET-results	Sensitivity	Specificity	Positive predictive value	Negative predictive value	Accuracy
	FDG	As positive	94% [70-100]	71% [57-83]	50% [39-61]	97% [85-100]	76% [65-86]
All HDVs (n=68)		As negative	94% [70-100]	75% [61-86]	54% [41-65]	98% [85-100]	79% [68-88]
	FLT	As positive	81% [54-96]	92% [81-98]	76% [55-90]	94% [85-98]	90% [80-96]
		As negative	69% [41-89]	96% [87-100]	85% [58-96]	91% [80-96]	90% [80-96]
cRT-HDVs (n=33)	FDG	As positive	93% [68-100]	61% [36-83]	67% [52-78]	92% [62-99]	76% [58-89]
		As negative	93% [68-100]	67% [41-87]	70% [54-82]	92% [64-99]	79% [61-91]
	FLT	As negative	80% [52-96]	94% [73-100]	92% [64-99]	85% [67-94]	88% [72-97]
		As positive	67% [38-88]	100% [81-100]	100%	78% [64-88]	85% [68-95]
SBRT-HDVs (n=35)	FDG	As positive	100% [3-100]	76% [59-89]	11% [6-19]	100%	77% [60-90]
		As negative	100% [3-100]	79% [62-91]	13% [7-22]	100%	80% [63-92]
	FLT	As positive	100% [3-100]	91% [76-98]	25% [10-50]	100%	91% [77-98]
		As negative	100% [3-100]	94% [80-99]	33% [12-68]	100%	94% [81-99]

cRT: conventional fractionated radiotherapy; SBRT: stereotactic radiotherapy

**Table 4:** Diagnostic value of FLT-PET/CT following positive FDG-PET/CT. 95% confidence interval in square brackets.

	Pretest probabili ty for malignancy*	Sensitivity	Specificity	Positive likelihood ratio	Negative likelihood ratio	Positive predictive value	Negative predictive value	Accuracy
All patients (n=44)	70%	74% [55-88]	77% [46-95]	3.2 [1.2-8.9]	0.3 [0.2-0.7]	88% [74-95]	56% [39-71]	75% [60-87]
cRT-patients (n=25)	72%	72% [47-90]	100% [59-100]	NA	0.3 [0.1-0.6]	100%	58% [40-75]	80% [59-93]
SBRT-patients (n=20)	70%	79% [49-95]	50% [12-88]	1.6 [0.7-3.7]	0.4 [0.1-1.5]	79% [61-90]	50% [22-78]	70% [46-88]
All HDVs (n=30)	50%	73% [45-92]	87% [60-98]	5.5 [1.5-20.7]	0.3 [0.1-0.7]	85% [59-95]	76% [58-89]	80% [61-92]
cRT-HDVs (n=21)	67%	71% [42-92]	100% [59-100]	NA	0.3 [0.1-0.7]	100%	64% [43-80]	81% [58-95]
SBRT-HDVs (n=9)	11%	100% [3-100]	75% [35-97]	4.0 [1.2-13.3]	0	33% [13-62]	100%	78% [40-97]

* Positive predictive value for positive/inconclusive FDG-PET/CT

cRT: conventional fractionated radiotherapy; SBRT: stereotactic radiotherapy; HDV: high-dose volume; NA: not applicable

## Key Points

### Question

Is FLT-PET valuable in diagnosing relapse of irradiated lung cancer?

### Pertinent Findings

FLT-PET/CT had a higher specificity and PPV than FDG-PET/CT on a patient-basis and within the irradiated high-dose volume.

### Implications

Adding FLT-PET to FDG-PET/CT when relapse is suspected in previously irradiated lung cancers improves diagnostic accuracy significantly.
