# Supplementary material for: 18F-FLT-PET/CT adds value to 18F-FDG-PET/CT for diagnosing relapse after definitive radiotherapy in patients with lung cancer. Results of a prospective clinical trial
Source: J Nucl Med. Author manuscript; Available in PMC 2021 Jul 25. (PMC7611370; doi:10.2967/jnumed.120.247742)
Supplement: Supplementary Material [file EMS130943-supplement-Supplementary_Material.pdf]

Supplemental Table 1: PET-models used for FDG-PET/CT

Hospital	PET manufacturer	PET model	No of patients
Herlev Hospital	Siemens	Biograph mCT 64	1
Rigshospitalet	Siemens	Biograph mCT 64	22
Rigshospitalet	Siemens	Biograph TruePoint TrueV 64	15
Rigshospitalet	Siemens	Biograph TruePoint TrueV 40	15
Bispebjerg Hospital	Philips Medical Systems	GEMINI TF TOF 64	4
Bispebjerg Hospital	GE Medical Systems	Discovery 710	4
Bispebjerg Hospital	GE Medical Systems	Discovery MI	2

FDG-PET/CT: 2-deoxy-2-[^{18}F]fluoro-D-glucose-PET/CT

SUPPLEMENTAL TABLE 2: FDG-PET/CT and FLT-PET/CT-results by clinical outcome within irradiated high-dose volume (HDV) (A-B) and on patient-basis (C-D). Results from blinded PET-evaluations. Numbers in brackets refer to subgroups (conventional fractionated radiotherapy/stereotactic radiotherapy).

A: HDV

		Reference standard		
		**Positive**	**Negative**	**Inconclusive**
	**Positive**	15 (14/1)	13 (6/7)	1 (1/0)
**FDG-PET/CT**	**Negative**	1 (1/0)	37 (11/26)	1 (0/1)
	**Inconclusive**	0	2 (1/1)	0

B: HDV

		Reference standard		
		**Positive**	**Negative**	**Inconclusive**
	**Positive**	11 (10/1)	2 (0/2)	1 (1/0)
**FLT-PET/CT**	**Negative**	3 (3/0)	48 (17/31)	1 (0/1)
	**Inconclusive**	2 (2/0)	2 (1/1)	0

C: Patient-basis

		Reference standard		
		**Positive**	**Negative**	**Inconclusive**
	**Positive**	31 (18/14)*	11 (6/5)	1 (1/0)
**FDG-PET/CT**	**Negative**	2 (2/0)	16 (6/10)	0
	**Inconclusive**	0 (0/0)	2 (1/1)	0

D: Patient-basis

		Reference standard		
		**Positive**	**Negative**	**Inconclusive**
	**Positive**	23 (13/11)*	3 (0/3)	1 (1/0)
**FLT-PET/CT**	**Negative**	7 (5/2)	22 (12/10)	0
	**Inconclusive**	3 (2/1)	4 (1/3)	0

*One patient was included in both subgroups

UPPLEMENTAL TABLE 3: Cross tabulations of FLT-PET/CT and FDG-PET/CT outcome in irradiated high-dose volumes (HDV) with relapse (A) and without relapse (B), and in patients with relapse (C) and without relapse (D). Results from blinded PET-evaluations. Numbers in brackets refer to subgroups (conventional fractionated radiotherapy/stereotactic radiotherapy).

A: HDV with relapse

		FDG-PET/CT		
		**Positive**	**Negative**	**Inconclusive**
**FLT-PET/CT**	**Positive**	11 (10/1)	0 (0/0)	0 (0/0)
	**Negative**	2 (2/0)	1 (1/0)	0 (0/0)
	**Inconclusive**	2 (2/0)	0 (0/0)	0 (0/0)

B: HDV without relapse

		FDG-PET/CT		
		**Positive**	**Negative**	**Inconclusive**
**FLT-PET/CT**	**Positive**	2 (0/2)	0 (0/0)	0 (0/0)
	**Negative**	9 (5/4)	37 (11/26)	2 (1/1)
	**Inconclusive**	2 (1/1)	0 (0/0)	0 (0/0)

C: patients with relapse

		FDG-PET/CT		
		**Positive**	**Negative**	**Inconclusive**
**FLT-PET/CT**	**Positive**	23 (13/11)*	0 (0/0)	0 (0/0)
	**Negative**	5 (3/2)	2 (2/0)	0 (0/0)
	**Inconclusive**	3 (2/1)	0 (0/0)	0 (0/0)

D: patients without relapse

		FDG-PET/CT		
		**Positive**	**Negative**	**Inconclusive**
**FLT-PET/CT**	**Positive**	2 (0/2)	0 (0/0)	1 (0/1)
	**Negative**	6 (5/1)	15 (6/9)	1 (1/0)
	**Inconclusive**	3 (1/2)	1 (0/1)	0 (0/0)

*One patient was included in both subgroups

Supplemental Table 4: Differences of sensitivity and specificity of FDG-PET/CT vs. FLT-PET/CT within the irradiated high-dose volume (HDV). Results from McNemar test. 95% confidence intervals in square brackets. *marks significant results.

	**Handling of inconclusive FDG-PET/CT**	**Handling of inconclusive FLT-PET/CT**	**Difference of sensitivity (percentage points)**	**p-value**	**n**	**Difference of specificity (percentage points)**	**p-value**	**n**
All HDVs	as positive	as positive	-13 [-29-4]	0.5000	16	21 [10-32]	0.0010*	52
		as negative	-25 [-46-(-4)]	*0.1250*	16	25 [13-37]	*0.0039**	52
	as negative	as positive	-13 [-29-4]	*0.5000*	16	18 [7-29]	*0.0002**	52
		as negative	-25 [-46-(-4)]	0.1250	16	21 [10-32]	0.0010*	52
cRT-HDVs	as positive	as positive	-13 [-31-4]	*0.5000*	15	33 [12-55]	*0.0313**	18
		as negative	-27 [-49-(-4)]	*0.1250*	15	39 [16-61]	*0.0156**	18
	as negative	as positive	-13 [-31-4]	*0.5000*	15	28 [7-48]	*0.0625*	18
		as negative	-27 [-49-(-4)]	*0.1250*	15	33 [12-55]	*0.0313**	18
SBRT-HDVs	as positive	as positive	0	*NA*	1	15 [3-27]	*0.0625*	34
		as negative	0	*NA*	1	18 [5-30]	*0.0313**	34
	as negative	as positive	0	*NA*	1	12 [1-23]	*0.1250*	34
		as negative	0	*NA*	1	15 [3-27]	*0.0625*	34

cRT: conventional fractionated radiotherapy; SBRT: stereotactic radiotherapy

Supplemental Table 5: Diagnostic value of FDG-PET/CT and FLT-PET/CT on patient-basis. Inconclusive PET-results were handled as positive, respectively, negative. Results from blinded PET-evaluations. 95% Confidence interval in square brackets.

Patients	PET	Handling of inconclusive PET result	Sensitivity	Specificity	Positive predictive value	Negative predictive value	Accuracy
All patients (n=62)	FDG	As positive	94% [80-99]	55% [36-74]	70% [61-78]	89% [67-97]	76% [63-86]
		As negative	94% [80-99]	62% [42-79]	74% [64-82]	90% [70-97]	79% [67-88]
	FLT	As positive	79% [61-91]	76% [56-90]	79% [66-88]	76% [61-86]	77% [65-87]
		As negative	70% [51-84]	90% [73-98]	88% [72-96]	72% [60-82]	79% [67-88]
cRT-patients (n=33)	FDG	As positive	90% [68-99]	46% [19-75]	72% [60-81]	75% [42-93]	73% [54-87]
		As negative	90% [68-99]	54% [25-81]	75% [62-85]	78% [46-93]	76% [58-89]
	FLT	As positive	75% [51-91]	92% [64-100]	94% [69-99]	71% [53-84]	82% [65-93]
		As negative	65% [41-85]	100% [75-100]	100%	65% [51-77]	79% [61-91]
SBRT-patients (n=30)	FDG	As positive	100% [77-100]	63% [35-85]	70% [55-81]	100%	80% [61-92]
		As negative	100% [77-100]	69% [41-89]	74% [58-85]	100%	83% [65-94]
	FLT	As positive	86% [57-98]	63% [35-85]	67% [51-80]	83% [57-95]	73% [54-88]
		As negative	79% [49-95]	81% [54-96]	79% [56-91]	81% [61-92]	80% [61-92]

cRT: conventionally fractionated radiotherapy; SBRT: stereotactic radiotherapy

Supplemental Table 6: Differences of sensitivity and specificity of FDG-PET/CT vs. FLT-PET/CT on patient level with variant handlings of inconclusive results. Results from McNemar test. 95% confidence intervals in square brackets. *marks significant results.

	**Handling of inconclusive FDG-PET/CT**	**Handling of inconclusive FLT-PET/CT**	**Difference of sensitivity (percentage points)**	**p-value**	**n**	**Difference of specificity (percentage points)**	**p-value**	**n**
All patients	as positive	as positive	-15 [-27-(-3)]	0.0625	33	21 [3-38]	_0.0703_	29
		as negative	-24 [-39-(-10)]	0.0078*	33	34 [17-52]	0.0020*	29
	as negative	as positive	-15 [-27-(-3)]	0.0625	33	14 [-5-32]	_0.2891_	29
		as negative	-24 [-39-(-10)]	0.0078*	33	28 [9-46]	_0.0215*_	29
cRT-patients	as positive	as positive	-15 [-31-1]	0.2500	20	46 [19-73]	_0.0313*_	13
		as negative	-25 [-44-(-6)]	0.0625	20	54 [27-81]	_0.0156*_	13
	as negative	as positive	-15 [-31-1]	0.2500	20	38 [12-65]	_0.0625_	13
		as negative	-25 [-44-(-6)]	0.0625	20	46 [19-73]	_0.0313*_	13
SBRT-patients	as positive	as positive	-14 [-33-4]	0.5000	14	0 [-17-17]	_1_	16
		as negative	-21 [-43-0.1]	0.2500	14	19 [-0.4-38]	0.2500	16
	as negative	as positive	-14 [-33-4]	0.5000	14	-6 [-27-15]	_1_	16
		as negative	-21 [-43-0.1]	0.2500	14	13 [-11-36]	_0.6250_	16

cRT: conventional fractionated radiotherapy; SBRT: stereotactic radiotherapy
